# In Vitro Wound-Healing Properties of Water-Soluble Terpenoids Loaded on Halloysite Clay

**DOI:** 10.3390/pharmaceutics13081117

**Published:** 2021-07-22

**Authors:** Lisa Marinelli, Ivana Cacciatore, Piera Eusepi, Marilisa Pia Dimmito, Annalisa Di Rienzo, Marcella Reale, Erica Costantini, Ana Borrego-Sánchez, Fátima García-Villén, César Viseras, Gianluca Morroni, Simona Fioriti, Lucia Brescini, Antonio Di Stefano

**Affiliations:** 1 Department of Pharmacy, University “G. d’Annunzio” of Chieti-Pescara, 66100 Chieti, Italy; ivana.cacciatore@unich.it (I.C.); piera.eusepi@unich.it (P.E.); marilisa.dimmito@unich.it (M.P.D.); annalisa.dirienzo@unich.it (A.D.R.); antonio.distefano@unich.it (A.D.S.); 2 Department of Innovative Technologies in Medicine and Dentistry, University “G. d’Annunzio” of Chieti-Pescara, 66100 Chieti, Italy; marcella.reale@unich.it; 3 Department of Medical, Oral and Biotechnological Sciences, University “G. d’Annunzio”, 66100 Chieti, Italy; erica.costantini@unich.it; 4 Department of Pharmacy and Pharmaceutical Technology, Faculty of Pharmacy, University of Granada, 18071 Granada, Spain; anaborrego@iact.ugr-csic.es (A.B.-S.); fgarvillen@ugr.es (F.G.-V.); cviseras@ugr.es (C.V.); 5 Andalusian Institute of Earth Science, CSIC—University of Granada, 18100 Armilla, Spain; 6 Department of Biomedical Sciences and Public Health, Polytechnic University of Marche, 60121 Ancona, Italy; g.morroni@univpm.it (G.M.); s.fioriti@pm.univpm.it (S.F.); lucia.brescini@ospedaliriuniti.marche.it (L.B.)

**Keywords:** clay, halloysite, skin regeneration, terpenoids, wound healing

## Abstract

Recently, mineral healing clays have gained much attention for wound-dressing applications. Here, we selected halloysite (HAL) clay as a biocompatible, non-toxic material that is useful as a drug delivery system to enhance the healing properties of water-soluble terpenoids 1-3 (**T1-3**). Terpenoids-loaded HAL clay (**TH1-3**) was prepared and characterized by adsorption equilibrium studies, X-ray powder diffraction (XRPD), thermogravimetric analysis (TGA), differential scanning calorimetry (DSC), Fourier-transform infrared (FTIR) spectroscopy, and release studies. The results reveal that **T1-3** were adsorbed at the HAL surface with good efficiency. The prevalent mechanism of drug retention is due to the adsorption via electrostatic interactions between the cationic groups of the **T1-3** and the HAL’s external surface. Release studies demonstrated that **T3** was released in a higher percentage (>60%) compared to **T1-2** (≈50%). Additionally, **TH1-3** were assessed for their antimicrobial activity and capability to promote the re-epithelialization of scratched HaCat monolayers, through the time-kill test and the wound-healing assays, respectively. The results reveal that all the tested formulations were able to reduce the microbial growth after 1 h of incubation and that they ensured complete wound closure after 48 h. Furthermore, at the concentration of 1 µg/mL, **TH3** exhibited 45% wound closure at 24 h, compared to **TH1** (27%) and **TH2** (30%), proving to be the best candidate in making the tissue-repair process easier and faster.

## 1. Introduction

The manipulation of drugs to produce nanoscale systems is finalized to overcome some drug physico-chemical properties such as taste, odor, stability, and solubility [1]. Nanosystems offer the possibility to modify the rate, time, or site of the drug release and prevent or reduce side effects [2]. Mineral clays are natural, abundant, low-cost, environmentally friendly, and non-toxic materials able to intercalate and/or adsorb specific molecules in their structure to achieve a drug delivery system with technological benefits [3,4]. In this field, halloysite (HAL), an aluminosilicate clay of the kaolin group, was extensively used as carrier of drugs for its advantageous nanosized tubular structure of around 600–800 nm in length and ~0.1 μm in external diameter [5,6]. Morphologically, HAL consists of a rolled bilayer of an outer negatively charged tetrahedral silicate sheet and an inner positively charged octahedral aluminum sheet, which offer the possibility of neutralization with different chemical entities [7]. HAL was investigated as an inorganic material able to retain essential oils, particularly carvacrol (CAR), a phenolic monoterpene with various biological and pharmacological properties [8]. Application of HAL is increasing in various biomedical fields and in the delivery of drugs [9,10]. CAR loaded into HAL lumen prevented CAR evaporation, sustained its release over time, and proved effective against *Escherichia coli*, *Pseudomonas putida*, *Listeria monocytogenes*, *Staphylococcus aureus*, *Aeromonas hydrophilia*, *Alternaria alternate*, and *Listeria innocua* [11,12,13]. Thus, these studies emphasized the importance of clay minerals, as CAR stability enhancer and drug delivery systems with potential application in the food packaging, hygiene, and personal care industries, as they are able to preserve the antimicrobial activity of the loaded compound. Furthermore, among different applications, HAL has gained much attention as wound-dressing material due to its mechanical features, cytocompatibility, and hemostatic and wound-healing properties.

**Figure 1:**
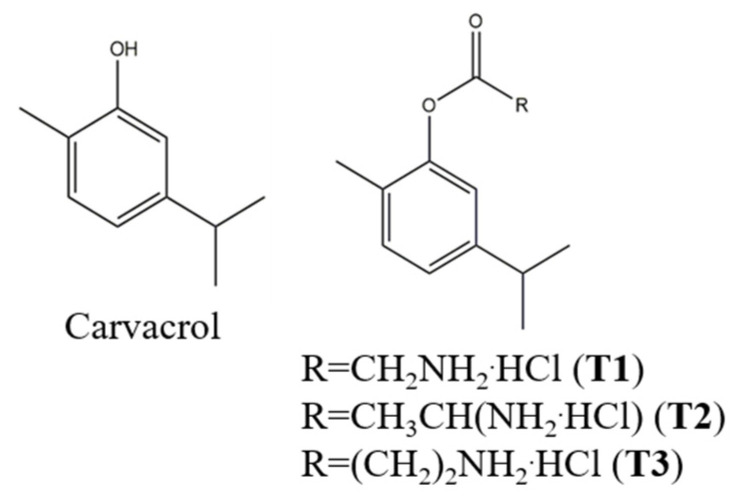
Carvacrol and **T1-3** structures.

Terpenoids 1-3 (**T1-3**) are prodrugs obtained by coupling amino acids such as glycine, L-alanine, and β-alanine (Figure 1) to CAR. They showed antimicrobial activities and increased water solubility compared to CAR (Table 1) [14]. In our previous works, hybrids of **T1-3** and layered montmorillonite or fibrous palygorskite and sepiolite were formulated to obtain their sustained release in the target site [15,16].

In this work, **T1-3**-loaded HAL (**TH1-3**) was developed and subjected to solid-state characterization, in vitro release studies, wound-healing, and antimicrobial assays to evaluate their potential as formulations for the treatment of wound infections that are otherwise difficult to treat.

## 2. Materials and Methods

### 2.1. Materials

Terpenoids 1-3 (**T1-3**) were synthesized as previously reported [14], and halloysite was purchased from Sigma-Aldrich (Merck KGaA, Darmstadt, Germany). All other materials employed were of analytical or HPLC grade.

### 2.2. Adsorption Equilibrium Studies

**TH1-3** nanocomposites were prepared following an adsorption procedure. In detail, a water solution of **T1-3** was placed in contact with HAL powder using a clay/drug ratio of 1:1 (*w/w*). The clay/drug suspensions were stirred in a water-shaking bath (Memmert GmbH + Co.KG, Schwabach, Germany) to achieve adsorption equilibrium. After centrifugation at 10,000 rpm, the solid hybrids were recovered and dried for subsequent investigations. To quantify the amounts of **T1-3** retained by the HAL powder, supernatants were examined as previously reported by High-Performance Liquid Chromatography (HPLC) [15,17].

### 2.3. Solid-State Characterization

#### 2.3.1. X-ray Powder Diffraction (XRPD)

Samples were examined on a Philips^®^ X-Pert diffractometer r (X’Pert3 MRD, Malvern, Cambridge, UK) between an exploration range of 4–70 2θ with CuKα radiation. The diffraction data were analyzed with XPOWDER^®^ software.

#### 2.3.2. Thermal Analysis

Thermogravimetric Analysis (TGA) and Differential Scanning Calorimetric (DSC) analyses were performed employing a METTLER TOLEDO mod (Mettler Toledo, Columbus, OH, USA). The TGA/DSC1 calorimeter combined with a FRS5 sensor and a microbalance (precision 0.1 μg) (Mettler Toledo, Columbus, OH, USA) were used for heating samples, in air atmosphere, at the rate of 10 °C/min. The temperature ranges employed were 30–950 °C and 30–400 °C for TGA and for DSC, respectively.

#### 2.3.3. Fourier Transform Infrared (FTIR)

FTIR analyses were performed in the wavenumbers ranging from 400 to 4000 cm^−1^, at 0.25 cm^−1^ resolution, using a JASCO 6200 FTIR spectrophotometer (JASCO 6200, JASCO International Co., Easton, MD, USA) coupled with an attenuated total reflectance (ATR) accessory and SPECTRA MANAGER v2 software (JASCO 6200, JASCO International Co., Easton, MD, USA).

### 2.4. In Vitro Release Studies

The desorption studies were performed by dispersing **T1-3**-loaded HAL formulations in a centrifuge tube containing 20 mL of a release medium composed of a phosphate buffer solution (PBS, pH 7.4). The dispersion was maintained at 37 °C in a water bath and was continuously stirred at 100 rpm using a magnetic bar. At predetermined times, the release kinetic was stopped and the tube was centrifuged at 10,000 rpm for 5 min; 1 mL of the supernatant was collected, filtered (0.45 μm, Millipore© filters), and analyzed by HPLC. The withdrawn release medium was replaced by an equal volume of fresh PBS. Calibration curves were drawn using **T1-3** to define the amount of drug released.

### 2.5. In Vitro Wound-Healing Assays

Human keratinocytes from adult skin (HaCaT cells) were seeded at a density of about 1.5 × 10^6^ cells/well (in 6-well plate) in Dulbecco’s modified Eagle’s medium supplemented with 10% heat-inactivated fetal bovine serum (FBS), 100 U/mL of penicillin, 100 µg/mL of streptomycin, and 2 mM L-glutamine (Merck KGaA, Darmstadt, Germany) at 37 °C in a 5% CO_2_ (*v*/*v*) humidified incubator and grown for 24 h to allow them to reach about 90% confluence. Then, cells were serum starved overnight. The monolayer of synchronized cells was gently scratched across the center of the well with a sterile pipette tip (Ø = 0.1 mm). After scratching, debris were removed by washing in PBS. Fresh medium containing 10% *v*/*v* of heat-inactivated FBS and treatments were added to each well. Images were obtained from the same fields immediately after scratching (t_0_) and after 24 and 48 h, using a Leica DMIL inverted microscope (Zeiss, Jena, Germany), and analyzed using ImageJ software by manually selecting the wound region and recording the total area. The experiments were conducted in triplicate, and two fields were analyzed for each replicate (*n* = 6). Untreated scratched cells represented the spontaneous. The percentage of cell-free area for each experimental condition was calculated relative to t_0_ = 100 cell-free area and Student’s T-test was used to evaluate the statistical significance (*p* < 0.05).

### 2.6. Time-Kill Assays

Time-kill assays were performed using methicillin-resistant *S. aureus* ATCC 43300, *S. aureus* ATCC 29213, *Staphylococcus epidermidis* ATCC 35984, and *Pseudomonas aeruginosa* ATCC 27853. Briefly, strains were diluted in 1 mL of cation-adjusted Mueller–Hinton broth to reach a final concentration of ~1 × 10^7^ CFU/mL and then incubated at 37 °C with 10 mg of HAL, **TH1**, **TH2,** and **TH3**. Serial dilutions at 1, 2, 4, and 8 h after initial inoculum were seeded in Mueller–Hinton agar or MacConkey agar (for staphylococci and *Pseudomonas*, respectively) to count CFU.

## 3. Results and Discussion

### 3.1. TH1-3 Characterization

**Figure 2:**
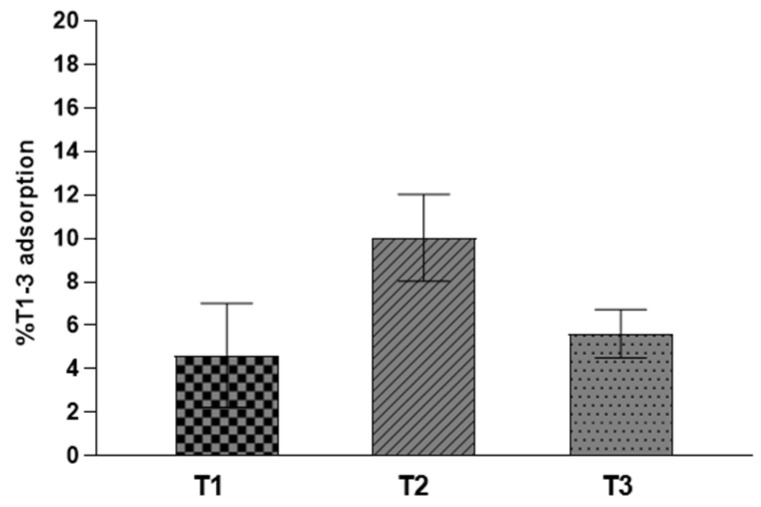
Percentage of **T1-3** adsorbed into HAL. Experiments are reported as mean and error bars represent standard deviations.

The adsorption procedure was carried out following the methodology described in paragraph 2.2. HAL, characterized by a positively charged internal surface and an external negatively charged shell, offers different methods to depot drugs. Organic molecules could interact with clay nanotubes by adsorption onto the clay surface, intercalation into interlayer spaces causing their expansion, and finally by entrapment into HAL lumens [18,19,20,21]. It was estimated about 5 mg of **T1** and **T3** and 10 mg of **T2,** for 100 mg of HAL, were effectively retained by the nanotubular powder (Figure 2). Lower measures of drug retention by HAL, than ones calculated for laminar and fibrous clays [13,14], were in accordance with HAL loading efficiency values ranging between 5 and 13%, reported for several drugs [7].

**Figure 3:**
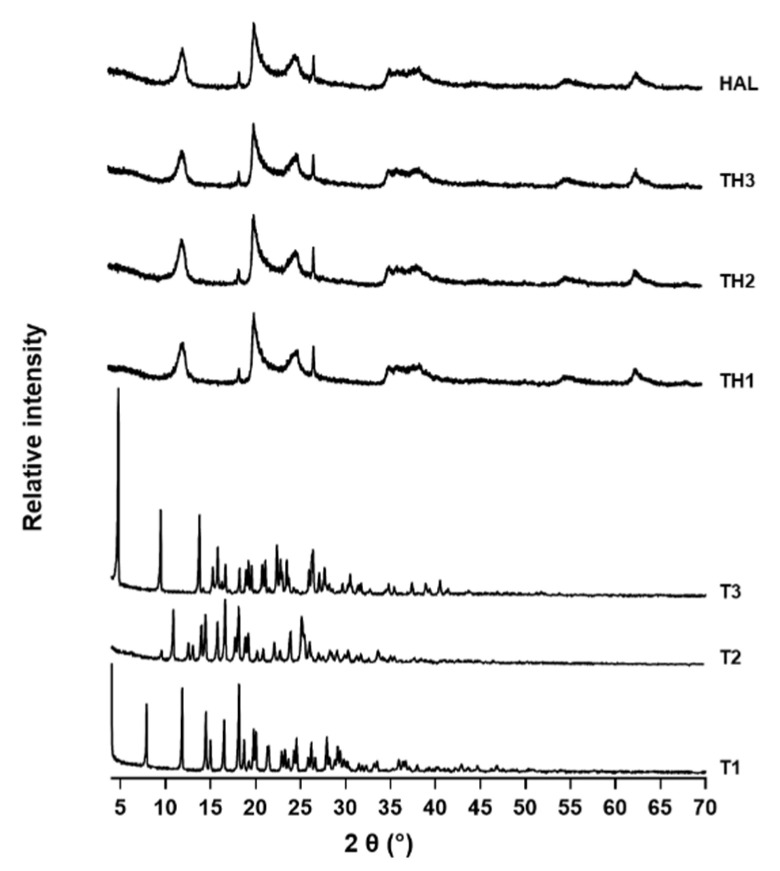
XRD patterns of HAL, **T1-3**, and **TH1-3**.

In Figure 3, the X-ray diffraction (XRD) pattern of **T1-3**-loaded HAL and precursor materials are compared. HAL shows major reflections at 12.2, 20.2, and 24.9 °2θ with basal spacing (d) of 7.2, 4.4, and 3.6, respectively Å confirmed the dehydrated state of Halloysite-7A and its tubular structure [22]. Additional sharp reflections at 26.8 °2θ with d = 3.3 Å and at 18.5 °2θ with 4.8 Å were typical of quartz and gibbsite impurities [19]. The loaded -HAL samples show the same characteristic peaks of HAL and its impurities, suggesting that **T1-3** did not modify HAL structures, but the absorption seemed to be limited to the external surface, as suggested also by no shifts to a lower angle of the diffraction peak at d001 [23,24].

**Figure 4:**
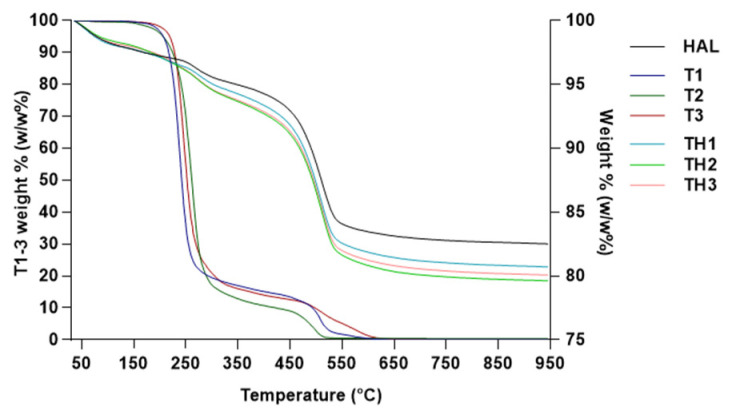
TGA curves of HAL, **T1-3**, and **TH1-3**.

Thermal behavior under increasing temperatures of **TH1-3**, raw HAL, and free **T1-3** are reported in Figure 4. In unloaded HAL, three-phase weight loss can be observed. The first mass loss between 50 and 200 °C of about 2.2% *w/w* was associated with the evaporation of the physically adsorbed water on the surface. The second mass loss of 1.3% *w*/*w* was detected in the temperature range of 200–380 °C and it was related to the evaporation of hydrogen-bonded water into the interlayer space. The last weight loss of 12.3% was detected up to 400 °C and it was correlated with the dehydroxylation process of the structural Al_2_OH [22,23,25]. HAL can be considered an extremely thermostable material since at the end of the analysis, the amount of HAL residue was 82.5% *w*/*w*, while **T1-3** underwent a total thermal decomposition under the experimental conditions. In **TH1-3,** weight losses were in correspondence to those of HAL in the intervals 50–180 °C, 180–380 °C, and up to 400 °C, but the degree of decomposition was greater than that observed in the HAL thermal profile. Hybrid TGA curves underlay the effective **T1-3** retention into the HAL structure. It was proposed that organic molecules preferentially arranged on the surface due to electrostatic attractions with the outer layer and repulsion with the inner layer [26,27].

**Figure 5:**
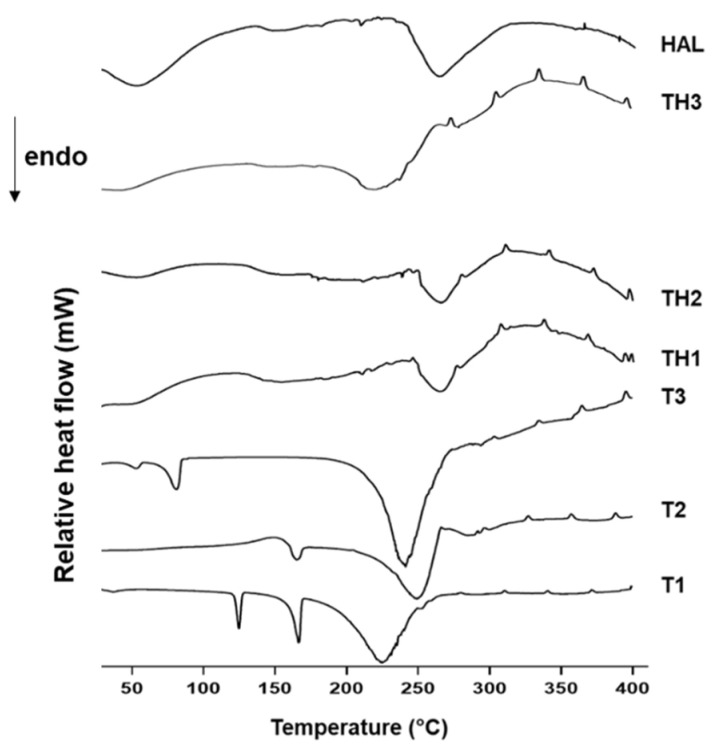
DSC curves of HAL, **T1-3**, and **TH1-3**.

In Figure 5, DSC profiles of pure HAL, **T1-3,** and their interaction products were compared. Unloaded HAL showed two endothermic peaks at 55 and 260 °C, corresponding to the loss of the physically adsorbed and intercalated water, respectively [28]. Loaded samples (**TH1-3**) did not show significant enthalpy energy deviations compared to the HAL profile, whereas the characteristic peaks of thermal degradation, present in the free prodrugs, completely disappeared, or became very low in intensity.

**Figure 6:**
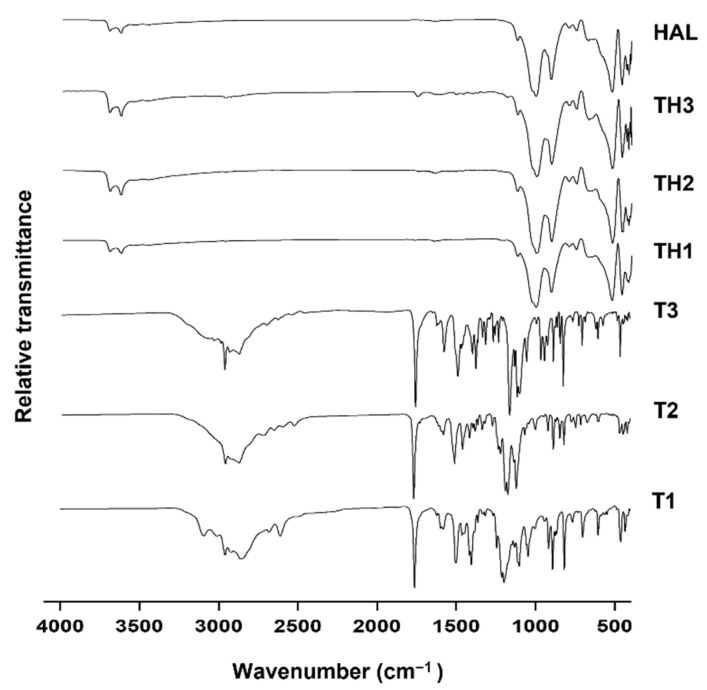
FT-IR pattern of HAL, **T1-3**, and **TH1-3**.

The interaction mechanism between organic and inorganic components was evaluated with FT-IR analysis, comparing the spectra of loaded nanotubes with untreated materials (Figure 6). Unloaded HAL showed distinctive bands at 3694–3623 cm^−1^ and 1652 cm^−1^ assigned to the stretching vibration of the two Al_2_OH and to the bending vibration of the physically adsorbed water, respectively. The observed band at 1119 cm^−1^ was generated by apical Si-O stretching vibration, those at 1003 and 907 cm^−1^ by Si-O-Si perpendicular stretching vibration, and those at 796 and 749 cm^−1^ to -OH translation vibration of HAL-OH units [17,19,29]. In nanohybrids, the presence of signals related to the inorganic and organic constituents mentioned above could suggest possible interactions between **T1-3** derivatives and nanotubes, without affecting the HAL structure. Nanohybrids presented characteristic bands associated with prodrugs and clay that slightly moved when compared to the pure materials. These shifts could suggest the formation of new Van der Waals forces, ionic interactions, and hydrogen bonding responsible for the retention process. Finally, in accordance with the XRPD study, the retention seems to be limited only to the external surface because the typical bands at 3674 and 3654 cm^−1^, visible only under a noise level of 0.1 and attributed to the stretching and out-of-phase vibrations related to the inner hydroxyl groups, did not disappear. Since the low amount of organic material in the nanocomposites, the X-array diffractogram patterns, and FT-IR results cannot alone confirm the successful interactions of the drug onto the HAL surface. These results become consistent if associated with the thermal analysis, which clearly highlights the effective adsorption between **T1-3** and HAL.

The reason for which **T1-3** do not reach the inner lumen should be of electrostatic nature: cationic prodrugs are rejected by the positively charged inner surface [30].

**Figure 7:**
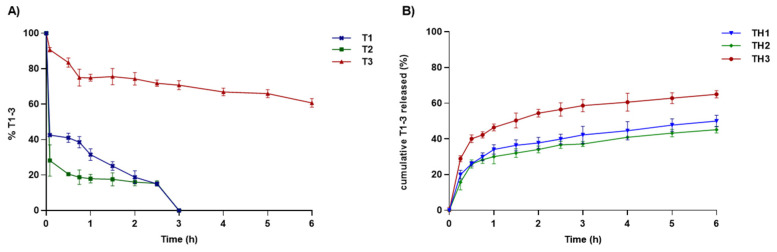
Percentage of **T1-3** after incubation for 6 h (**A**), and cumulative % of **T1-3** released from nanocomposites **TH1-3** (**B**). Results are expressed as mean of three experiments and error bars represent the standard deviations.

HAL-based nanocomposites were developed to improve drug-like properties as well as the bioavailability and efficacy of **T1-3**. These goals have been achieved, as shown in the release profiles reported in Figure 7. Compared to the free **T1-3**, all the formulations ensure a slower release of **T1-3** over time, protecting them from unfavorable environmental conditions that favor their premature hydrolysis. The rate of drug release is greater for **TH3** (>60%) compared to **TH1-2** composites. Since the proposed wound-healing application, the ability to control the drug release could be useful for better wound management. Indeed, the sustained release offers the advantage to reduce the frequencies of administration and guarantees a continuous level of therapeutics in the site of action, which gradually, at physiological pH, undergo bioconversion in the parent drugs by hydrolysis of the ester linkage. After 6 h, **T1-3** were not completely released from HAL nanocomposites, probably because during the desorption processes, drug–clay interactions prevail over those between the clay and ions present in the external medium.

Taking together these results, it was proposed that **T1-3**, due to their physico-chemical features, interact with the clay and exploit an adsorption via electrostatic interaction between the **T1-3** cationic amine group and the HAL-external negatively charged surface. This prevalent mechanism of drug retention, as well as the interaction processes, were confirmed through the solid-state characterization of drug–clay hybrids, which proves the effective retention of **T1-3** into the HAL structure. Furthermore, release studies revealed the suitability of **TH1-3** as a sustained delivery strategy useful to guarantee therapeutic levels of drugs to the site of action and ensure their subsequent penetration in infected wounds.

### 3.2. Wound Healing and Antimicrobial Assays

**Figure 8:**
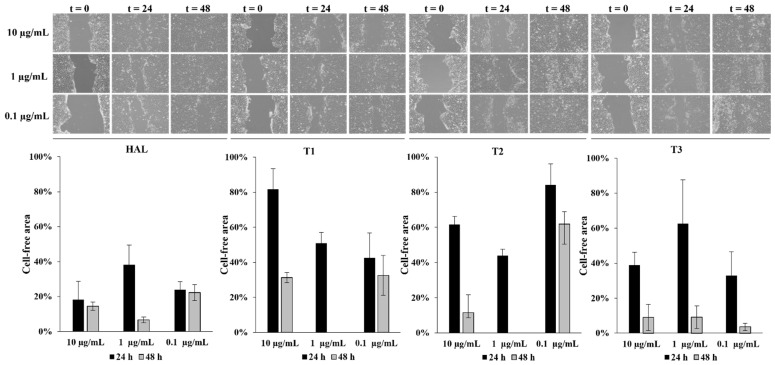
Dose–response and time course effect of **T1-3** on wound healing. Micrographs show representative results of a wound immediately after the scratches (t_0_), and after 24 and 48 h in the presence of **Ts**. Wound closure was evaluated by measuring the remaining cell-free area and expressed as a percentage of the initial cell-free area. The results of three independent experiments are expressed as mean ± SD of percentage of the cell-free area.

To understand the effect of **TH1-3** in the re-epithelialization and wound healing, cell-based scratch assays with a HaCaT cells monolayer were performed on an in vitro artificial wound model. Preliminary experiments were performed using different concentrations (10, 1, 0.1 µg/mL) of HAL and **T1-3**. The rate of scratched monolayer closure was evaluated through the observation of the re-population in the area between the wound edges after 24–48 h from the induced injury. Optical microscopy was used to observe cells migrating into the wound area and a series of images were acquired, within the range 24–48 h, to study the cells’ migration into the cell-free gap (assumed 100% at time zero), and the wound closure. Results, from three independent experiments in duplicate, expressed as reduction in free-cells area percentages, as well as representative images from microscopic observations of scratched monolayers, are shown in Figure 8.

**Figure 9:**
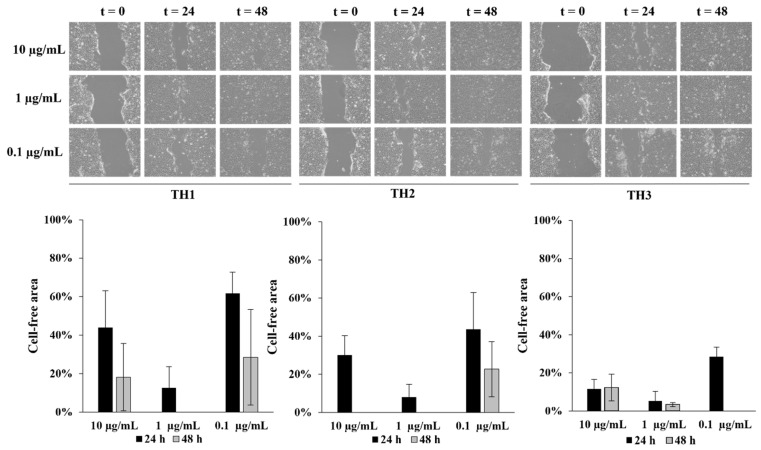
Dose–response and time course effect of **TH1-3** on wound healing. Micrographs show representative results of the wound immediately after the scratches (t_0_), and after 24 and 48 h in the presence of **TH1-3**. Wound closure was evaluated by measuring the remaining cell-free area and expressed as a percentage of the initial cell-free area. The results of three independent experiments are expressed as mean ± SD of percentage of cell-free area.

**Figure 10:**
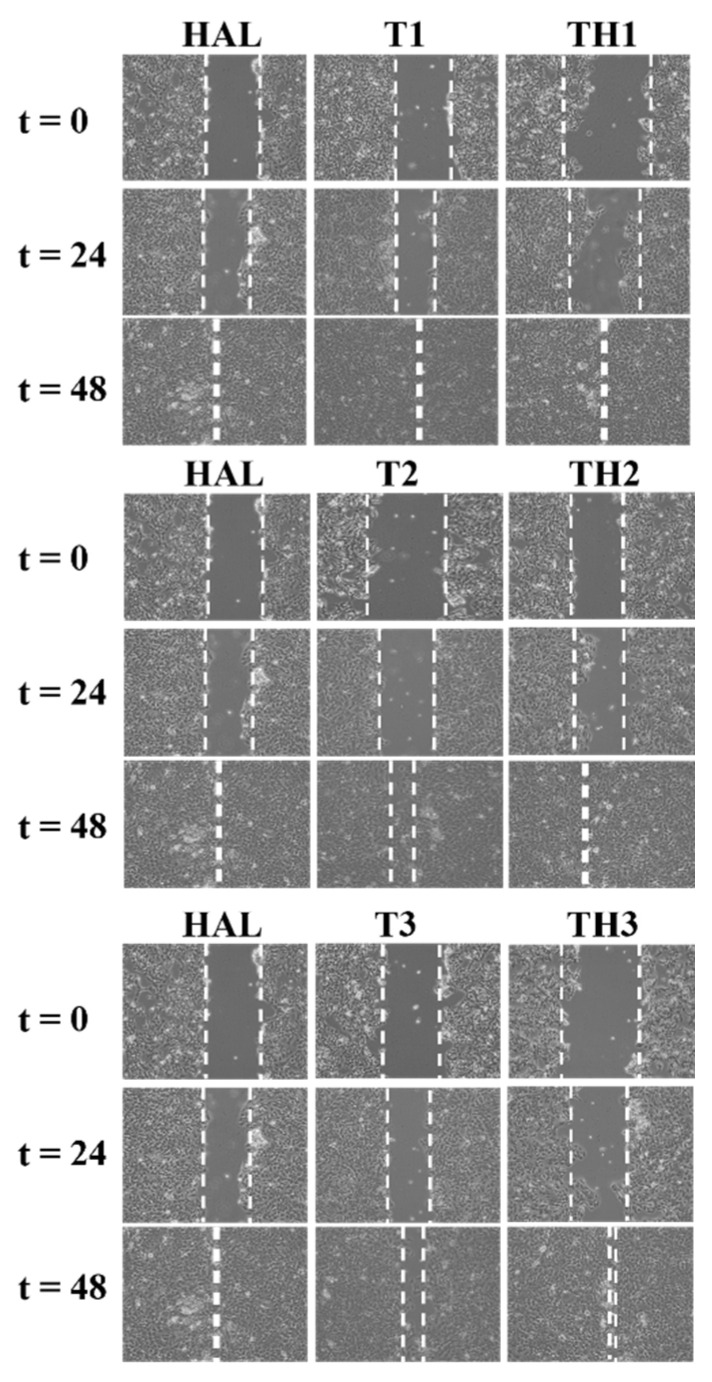
Time-dependent effects of HAL, **T1-3**, and **TH1-3** at 1 μg/mL on wound healing. Representative images of wound closure assays after HAL, **T1-3**, or **TH1-3** treatment for up to 48 h. The wound-healing width were marked by the manual drawing of a line at the wound edges on each image, obtained from three different experiments.

After 24 h of scratching, the cell-free area, exposed to 1 µg/mL of HAL, **T1**, **T2**, and **T3**, decreased from 100% to 38%, 51%, 44%, and 63%, respectively. HAL, at all the tested concentrations, improved wound closure, showing the best efficacy after 48 h, at the assessed concentration of 1 µg/mL (cell-free area: 7%). After 48 h from the wound induction, **T1** and **T2**, at the concentration of 1 µg/mL, promoted a complete closure of the wound area while, in cells treated with **T3**, 9% of the wound was cell-free. At 24 h, **THs** formulations, at 1 µg/mL, induced a significant cell migration, reducing the gap size up to 13%, 8%, and 5% for **TH1**, **TH2**, and **TH3**, respectively. At 48 h, a complete wound closure (0% cell-free area) was achieved (Figure 9). Based on these considerations, the concentration of 1 µg/mL was selected as the best one. Figure 10 better elucidates the effects of 1 µg/mL of HAL, **T1-3,** and **TH1-3** on the repair process. All the tested formulations improved the closure of the open wound better than HAL or **T1-3** separately. These data are also summarized in Table 2. In fact, 24 h after the injury, the treatment with **TH1-3** significantly accelerated the rate of the monolayer repair process compared to HAL or **T1-3** alone. **TH1** improved the wound healing, reducing the wound width up to 27% compared to the 30% and 45% observed with **TH2** and **TH3**, respectively (Table 2). **TH1-3** favored the re-epithelialization of scratched HaCat monolayers more than free **T1-3**. Furthermore, **TH3** at 1 µg/mL has greater biological activity on wound healing and is more efficacious than **TH1** and **TH2** at the same tested concentration. The analysis revealed not significant differences between spontaneous and HAL alone, **T1-3** and **TH1**, while a significant increase in closure rate was observed in the presence of **TH2** and **TH3**. No significant variations were observed comparing **T1-3** and **TH1-3** to HAL alone. After 48h, the percentage of free area was reduced in all samples, thus no significant differences between all conditions were observed. Bearing in mind the essential barrier function of the epidermis, when damage happens, it is necessary to re-establish tissue integrity quickly and as best as possible through the re-epithelialization process. Keratinocyte proliferation and migration represent essential steps in the re-epithelialization process during wound healing. The higher activity of **TH1-3**, compared to the free **T1-3**, can be attributed to the nano size structure, as well as to the high surface area-to-volume ratio. It is likely that these features allow the nanocomposites to establish additional favorable interactions with the wound area, ensuring an easier penetration of the skin layers.

**Figure 11:**
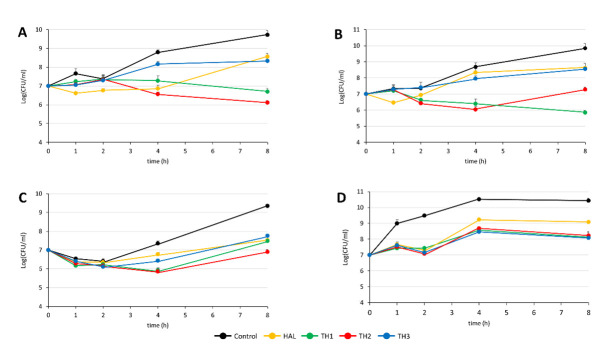
Time-kill curves of HAL and **TH1-3** (10 mg/mL) on different bacterial strains: (**A**) *S. aureus* ATCC 43300; (**B**) *S. aureus* ATCC 29213; (**C**) *S. epidermidis* ATCC 35984; (**D**) *P. aeruginosa* ATCC 27853.

Exposed tissues in wounds are more susceptible to microbial contamination, which can produce detrimental alterations and subsequent impairment in the wound-healing processes [31]. Notably, aerobic, or facultative pathogens are the major triggers of delayed healing and infections in chronic and acute wounds. Considering that the healing process could be affected also by the presence of bacteria, **TH1-3** formulations were further evaluated for their antimicrobial activity against *S. aureus*, *P. aeruginosa*, and *S. epidermidis*. *S. aureus*, and *epidermidis* were selected as prototypes of Gram-positive and negative bacteria, respectively, and are notoriously resistant to therapeutics, which frequently colonize chronic wounds [32,33,34]. The choice of the selected microorganisms also took into account their prevalence in the various stages of the wound. Indeed, there is evidence that in an early phase of the chronic wound, *S. aureus*, including methicillin resistant *S. aureus* (MRSA), are predominant, while, in the advanced stages, Gram-negative bacteria, including *Pseudomonas* varieties, are recognized as responsible for a penetration of the profounder layers of skin, producing significant tissue injury [31,33]. In time-kill assays, a reduction in the CFU was observed after 8 h when bacteria were grown in the presence of HAL and **TH1-3**. **TH1** showed the greatest activity against *S. aureus*, with a decrease of 3 and 2 log compared to the control and unloaded HAL, respectively (Figure 11A). **TH2** had a visible effect on *S. aureus* ATCC 43300 but resulted as being less active against *S. aureus* ATCC 29213 (Figure 11B). Conversely, HAL alone and **TH1-3** showed similar effects against *S. epidermidis* (Figure 11C). A reduction of 2 log of the CFU were observed when the *P. aeruginosa* strain growth was treated with **TH1-3**, while HAL alone demonstrated a lower activity (Figure 11D). Moreover, the growth reduction was notable already after 1 h of incubation.

Our results highlight that **TH1-3** possess wound-dressing activity, combining tissue regeneration and antimicrobial properties, as suggested by both wound-healing and time-kill experiments. They are able to promote the re-epithelialization of scratched HaCat monolayers better than **T1-3** alone, making the tissue repair process easier. Furthermore, **THs**, in some cases, enhanced the HAL activity by affecting the bacterial growth. These results could be particularly interesting since treatment of wounds with **TH1-3** could reduce the time for the wound healing and at the same time hamper the bacterial growth, protecting wounds from infections or blocking a contamination that is already in progress, with considerable advantages for the patients.

## 4. Conclusions

Wound management has gained much interest in recent decades due to the increase in chronic wounds and the complications correlated with them. In this context, the present work describes the development of potential wound-dressing materials obtained by combining halloysite nanocomposites as a drug delivery system, and **T1-3** as antimicrobials. Based on the performed characterization studies, all the formulations provide a controlled release of the adsorbed compounds. In vitro studies highlighted that **TH1-3** possessed greater wound-healing properties than the free **T1-3**, as well as antimicrobial properties. Specifically, **TH2** and **TH3** demonstrated a significantly greater ability to promote scratch repair compared to spontaneous wound healing (*p* = 0.03; *p* = 0.02, respectively), suggesting their potential use as wound-dressing material. These data support further investigations of the developed formulations in an in vivo model of infected wounds.

## Figures and Tables

**Table 1:** Drug-like properties of CAR and **T1-3**.

	CAR	T1	T2	T3
Molecular Weight (g/mol)	150.22	243.73	257.76	257.76
Water Solubility (mg/mL) ^a^	0.11	586.87	190.63	479.33
LogP ^b^	2.82	2.17	2.53	2.43

^a^ Experimentally calculated [14], ^b^ Prediction SwissADME platforms.

**Table 2:** Wound healing is expressed as the percentage of cell-free area (mean ± SD) relative to the original size of each treatments (wound area)/(original wound area)·100.

	Percentage Wound Width	
	T = 24 ^b^	T = 48 ^b^
Spontaneous	88 (±5)	16 (±6)
HAL	35 (±13)	0 (±7)
T1	58 (±17)	0 (±5)
T2	62 (±8)	14 (±3)
T3	62 (±12)	27 (±8)
TH1	73 (±17)	0 (±5)
TH2	70 (±8) *	0 (±3)
TH3	55 (±14) *	0 (±1)

^b^ Values are means of three experiments; standard deviation is given in parentheses; * *p* < 0.05, relative to spontaneous wound healing.

## Data Availability

Data are contained within the article.
